# Impact of a mobile unit on access to eye care in São Paulo, Brazil

**DOI:** 10.5935/0004-2749.20210009

**Published:** 2025-02-02

**Authors:** Larissa Horikawa Satto, Roberta Lilian Fernandes de Souza Meneghim, Flavio Eduardo Hirai, Carlos Roberto Padovani, Silvana Artioli Schellini

**Affiliations:** 1 Departamento de Oftalmologia, Otorrinolaringologia e Cirurgia de Cabeça e Pescoço, Faculdade de Medicina de Botucatu, Universidade Estadual Paulista “Julio de Mesquita Filho”, Botucatu, SP, Brazil; 2 Departamento de Oftalmologia, Universidade Federal de São Paulo, São Paulo, SP, Brazil; 3 Departamento de Bioestatística, Universidade Estadual Paulista “Julio de Mesquita Filho”, Botucatu, SP, Brazil

**Keywords:** Mobile health units, Eye health, Vision disorders, Refractive errors, Eyeglasses, Blindness/prevention & control, Unidades móveis de saúde, Saúde ocular, Transtornos da visão, Erros de refração, Óculos, Cegueira/prevenção & controle

## Abstract

**Purpose:**

The goal of this study was to determine the impact of a mobile eye health unit on access to eye care and to generate a profile of the population requiring ophthalmic care by age, nature of their ophthalmic diseases, and optimal management.

**Methods:**

The study was conducted in 14 cities in the southwest region of São Paulo, Brazil. Subjects included individuals who participate in the Brazilian Unified Health System who were in need of eye care. There were no restrictions on age, gender or socioeconomic status. Data was transferred to an Excel table for statistical analyses.

**Results:**

We evaluated 6,878 participants in this survey with mean age of 44 years (range 4 months to 96 years); 65.5% were female. Among the diagnoses, 78.6% presented with refractive errors, 9.6% presented with cataracts and 8.3% presented with pterygium. New corrective lenses were prescribed for 60.9% of the participants; 10% retained their existing lenses, ~28% required counseling only and18.1% of the participants were referred to a tertiary facility for specialized exams and/or surgical procedures. Of the participants who required outside referrals, 36.4% required oculoplastic/external eye surgery and 31.8% required cataract surgery.

**Conclusion:**

The vast majority of patients presenting to a mobile eye health unit required prescriptions for corrective lenses. The rate of detection of ocular disorders was relatively high and the mobile unit provided effective treatment of refractive errors and referrals for specialized ophthalmic examinations and procedures. A mobile eye health unit can be an effective alternative method for improving access to basic eye care, for promoting eye health education and preventing blindness.

## Abstract (pt)

**Objetivo:**

Determinar o impacto do uso de unidade móvel no acesso à saúde ocular e avaliar o perfil da população que necessita de cuidados oftalmológicos, as doenças oculares mais frequentes e o tratamento.

**Métodos:**

Estudo transversal realizado em 14 municípios da região sudoeste do Estado de São Paulo utilizando uma unidade móvel oftalmológica. Os participantes eram usuários do Sistema Único de Saúde que procuraram atendimento oftalmológico, sem restrição quanto a idade, gênero ou condição socioeconômica. Os dados foram transferidos para a tabela Excel para análise estatística.

**Resultados:**

Participaram do estudo 6.878 pessoas, com média de idade de 44 anos (variação de 4 meses a 96 anos) e 65,5% eram mulheres. Erros refrativos estavam presentes em 78,6% dos participantes, catarata em 9,6% e pterígio em 8,3%. Para 60% foram prescritos óculos, para 10% foi mantida a correção óptica em uso e para 28% foram necessárias apenas orientações. Exames especializados ou procedimentos cirúrgicos foram indicados para 18,1% dos casos que foram encaminhados para tratamento em serviço terciário. Dentre os pacientes referenciados, 36,4% necessitavam de cirurgia oculoplástica ou para tratar afecções externas do olho e 31,8%, de cirurgia de catarata.

**Conclusão:**

A grande maioria dos pacientes que procurou atendimento na unidade móvel necessitava de prescrição de óculos. A unidade móvel oftalmológica possui alto grau de resolutividade para os problemas oculares, com oportunidade de tratar os erros refrativos e referenciar os pacientes que necessitam de atendimento espe cializado, geralmente relacionado a condições cirúrgicas. Unidades móveis podem ser uma alternativa aos cuidados oftalmológicos básicos, melhorando o acesso, atuando na promoção da saúde ocular e prevenindo a cegueira.

## INTRODUCTION

Globally, there are 253 million visually-impaired individuals of whom 217 million experience moderate to severe dysfunction; 36 million individuals are legally blind^(1)^. It is estimated that 80% of the cases of visual impairment and blindness are caused by disorders that are either curable or preventable^(2,3)^.

The prevalence of visual impairment has decreased in the past 20 years, although the absolute number of cases has increased due to population growth and aging^(4)^. In Brazil, 18.8% of the population is visually impaired, including almost 50% of individuals over 65 years of age^(5)^; ~0.4 to 0.5% of the Brazilian population suffers from blindness^(6)^.

One key measure to reduce visual impairment and blindness is to provide effective access to eye care services^(2)^. Although the number of ophthalmologists has increased in Brazil to a level that is currently considered to be sufficient, availability and access to ophthalmic services are vastly different in different regions and do not meet the needs of all communities; this is mainly due to economic difficulties, poor distribution of resources and limited infrastructure^(7-9)^. This is of particular concern given that aging is clearly associated with a significant increase in the incidence of ophthalmic disorders^(4,7,8)^.

While eighty percent of the Brazilian population depends on public health care provided by the Unified Health System (SUS), only 25% of the practicing ophthalmologists participate in this system. As such, those who rely on the public system currently do not have effective access to eye care services^(10)^. Even when ophthalmic care is available, other factors can hinder access to eye care^(6)^, including inadequate transportation, limited social support and the comparatively high cost of evaluation and treatment^(11)^.

Brazilians have no access to eye care^(12)^. Mobile units represent a feasible alternative for those living in small municipalities and at the periphery of large cities and/ or for providing coverage for specific target populations. The mobile units can be equipped for basic ophthalmic examinations and should be capable of screening for more complex eye diseases and generating appropriate referrals for treatment^(13,14)^.

The model of mobile units was originally conceived in the United States early in the 20^th^ century and were put into practice as Community Mobile Eye Clinics; this model was ultimately used as a means to provide other medical services in addition to basic eye examinations^(15)^. These units were used primarily by those who had limited access to basic health care, primarily in resource-poor regions with inadequate physical infrastructure^(16)^.

Several countries are currently using mobile eye health units to improve access to eye care and have collected epidemiological data and generated screening programs in impoverished areas and among segments of the population who have limited access to health care^(17)^. Primary eye care provided by mobile units is typically more efficient at providing care for the population at large than a traditional practice^(14)^. However, the impact of mobile units on ocular health is difficult to evaluate quantitatively as there are substantial geographic and socioeconomic variations among those in the population to be served and likewise among the services offered^(17)^.

The purpose of this study was to determine the impact of a mobile eye health unit on access to eye care and evaluate the profile of the population requiring ophthalmic consultation in São Paulo, Brazil. We also identified the main ocular diseases in the community and their appropriate management in this setting.

## METHODS

This research was approved by the Ethics and Research Committee of the Faculty of Medicine of Botucatu, São Paulo State, Brazil and the study adhered to the tenets of the Declaration of Helsinki. All subjects underwent a thorough informed consent procedure and were required to sign an informed consent form prior to participation in the study.

The study was conducted in 2011 in the urban areas within 14 municipalities from the southwest region of São Paulo State, Brazil, and enrolled participants who spontaneously requested services from a mobile eye health unit in their municipality. Individuals of any age, gender or socioeconomic status were included as participants in the study. Individuals who refused to participate were excluded from this study.

**Figure 1:**
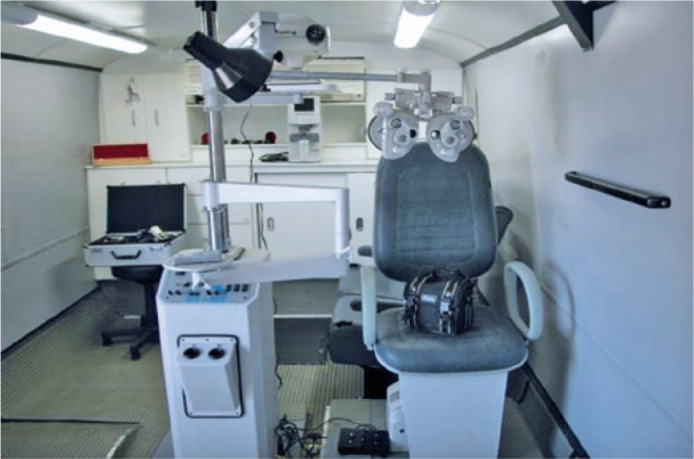
Mobile eye health unit: a bus that has been adapted to provide eye care.

The mobile eye health unit was a bus adapted for ophthalmic care (Figure 1). This bus was equipped with an auto lensometer (AL 500 Reichert, NY, USA), an auto refractor (Accuref K Shinn Nippon, Tokyo, Japan), a manual refractor, a retinoscope, skiascopic rulers, ‘E’ charts, a direct ophthalmoscope (Welch Allyn Inc., NY, USA), 78 diopter lenses (Volk Optical Inc., Ohio, USA), slit lamp (Shinn Nippon, Tokyo, Japan), a pneumo-tonometer (CT-60, Topcon, Tokyo, Japan) and an applanation tonometer (Goldmann tonometer Haag Streit, Switzerland).

Standardized eye examinations were performed by a trained team that included two ophthalmologists, three ophthalmology residents and four technicians who provided support services, including filling out forms, arranging patient flow, instilling eye drops and providing general information.

The order of the examination was determined by a specific protocol using demographic data, specific eye complaints, self-reported systemic or ocular diseases and a family history of eye problems. The ocular exam was divided into stations as follows: pre-consultation, visual acuity, pneumatic intraocular pressure, automatic objective refraction, pupillary dilation and/or cycloplegia, biomicroscopy and fundoscopy.

Uncorrected visual acuity for distance was evaluated for each eye using an illiterate ‘E’ chart placed six meters from the participant; a second test was performed with eyeglasses if in use. If the patient was unable to see the top line of the chart at six meters, the vision was tested and recorded as counting fingers, hand movements, light perception or no light perception. Children who were pre-verbal were evaluated by preferred gaze or light tracking. Based on the results of this preliminary examination, subjective refraction was performed for those with ocular complaints of reduced visual acuity or symptoms of asthenopia. If the participants were less than 40 years old, they underwent a cycloplegic refraction 30 minutes after instillation of cyclopentolate (Cicloplegic^®^, Allergan, Guarulhos/SP, Brazil). Biomicroscopic exam after instillation of three drops of mydriatic eye drops (Mydriacyl^®^, Alcon, São Paulo/SP, Brazil) within an interval of five minutes, and examination after 30 minutes was done to identify causes of low vision which did not improve with a refractive correction. Goldmann tonometry was performed for individuals >40 years old, in individuals with a family history of glaucoma, and in those with suspected glaucoma. Fundoscopic examination under mydriasis was performed for patients with hypertension, diabetes mellitus, visual impairment without improvement with refraction and for patients with high refractive errors.

The dDefinition of visual impairment and blindness was adopted from the tenth edition of the international code of diseases by World Health Organization (WHO) based on visual acuity (VA) as follows: moderate visual impairment >0.1 VA <0.3; severe visual impairment, >0.05 VA <0.1 and blindness VA <0.05^(18)^. The measure of VA was based on the results from the better of the two eyes after refraction.

After completion of the ophthalmic examination, an ophthalmologist determined whether corrective lenses were required and/or whether any additional clinical/ surgical treatments were warranted and required referral to a regional tertiary hospital.

The data were transferred to an Excel table for statistical analyses; p<0.05 was considered as statistically significant.

## RESULTS

The specific cities, their characteristics, and the num ber of study participants from each municipality are pre sented in table 1.

**Table 1:** Characteristics of the cities served by the mobile eye health units and and number of participants

City	Location^a^ Latitude Longitude	Distance to Botucatu^a^ (km)	IDHM^b^	Population^c^	Number of ophthalmologists^d^ SUS	Number of participants
Águas de Santa Bárbara	22°52ʹ 49°15ʹ	108	0.757	5,601	0 0	456
Assis	22°39ʺ42ʹ 50°24ʺ44ʹ	250	0.805	95,144	10 6	1,444
Barra Bonita	22°29ʺ41ʹ 50°24ʺ44ʹ	59.9	0.788	35,246	4 4	233
Bernardino de Campos	23°00ʺ47ʹ 49°28ʺ27ʹ	137	0.734	10,775	1 1	335
Botucatu	22°53ʺ09ʹ 48°26ʺ42ʹ	0	0.800	127,328	15 9/35^*^	1,222
Brotas	22°1ʺ72ʹ 48°7ʺ37ʹ	92,6	0.740	21,580	3 0	561
Dois Córregos	22°21ʺ58ʹ 48°22ʺ49ʹ	81.2	0.725	24,761	0 0	218
Maracaí	22°36ʺ39ʹ 50°40ʺ1ʹ	277	0.771	13,332	0 0	309
Óleo	22°56ʺ29ʹ 49°20ʺ31ʹ	128	0.730	2,673	0 0	355
Pratânia	22°48ʺ30ʹ 48°39ʺ58ʹ	38.4	0.701	4,599	0 0	208
Promissão	21°32ʺ12ʹ 49°51ʺ29ʹ	222	0.743	35.674	0 0	233
Taquarituba	23°31ʺ59ʹ 49°14ʺ40ʹ	138	0.701	22,291	1 1	799
Tarumã	22°44ʺ48ʹ 50°34ʺ38ʹ	271	0.753	12,885	0 0	208
Torrinha	22°25ʺ34ʹ 48°10ʺ09ʹ	73,5	0.744	9,330	0 0	297

* number including ophthalmologists of Ophthalmology Service of Botucatu Medical School

a= Google Earth Mapas [Internet]. [citado 2018 Abr 15]. Disponível em]: https://www.google.com/earth/

b= Atlas do desenvolvimento humano no Brasil 2013 [Internet]. Programa das Nações Unidas para o Desenvolvimento; 2013.[citado 2018 Abr 15]. Disponível em: http://www.atlasbrasil.org.br/2013/

c= Instituto Brasileiro de Geografia e Estatística(IBGE) [Internet]. Rio de Janeiro: IBGE; 2015 [citado 2018 Abr 15]. Disponível em: http://www.ibge.gov.br/home/estatistica/populacao/censo2010/tabelas_pdf/Brasil_tab_1_14.pdf

d= Brasil. Ministério da Saúde. Departamento de Informática do Sistema Único de Saúde - DATASUS - Cadastro Nacional dos Estabelecimentos de Saúde do Brasil - CNES [Internet]. Brasília (DF): CNES; 2015 [citado 2018 Abr 15]. Disponível em: http://tabnet.datasus.gov.br/cgi/tabcgi.exe?cnes/cnv/prid02sp.def.

The study included 6,878 participants. The mean age was 44 years old (range 4 months to 96 years). Of these, 4,508 (65.5%) were female.

The most common ocular complaints were reduced near VA which presented in 4,151(60.4%) of the participants followed by reduced far VA presented by 3,851 (56%) of the participants (Table 2).

**Table 2:** Main ocular complaints of the participants

Ocular complaint	Number of participants	%
Near visual difficulty	4,151	60.4
Far visual difficulty	3,851	56.0
Headache	1,836	26.7
Ocular pain	1,071	15.6
Pruritus	446	6.5
Hyperemia	365	5.3
Tearing/photophobia	212	3.1
Foreign body sensation	177	2.6
No complaint	176	2.6
Cataracts	106	1.6
Pterygium	82	1.2
Floaters/scotomata	70	1.0
Strabismus	24	0.4
Diplopia	17	0.2
Wounds and injuries	14	0.2
Secretions	12	0.2
Glaucoma	8	0.1
Edema	8	0.1
Blepharospasm	4	0.1
Color vision disturbances	4	0.1

* The same patient may have reported more than one complaint.

Based on participant response, 4,359 (63.4%) of the individuals were otherwise healthy; 2,151 (31.3%) had been diagnosed with hypertension and 797 (11.6%) with diabetes mellitus. Corrective lenses had been prescribed previously for 2,350 (34.2%); 341 (5%) had undergone cataract surgery, 271(3.9%) had undergone pterygium resection, 96 (1.4%) reported glaucoma and 84 (1.2%) reported previous ocular trauma.

The best corrected VA was within normal limits for 6,290 participants, or 92.4% of the study population. Another 349 participants (5.2%) had moderate and severe visual impairment and 134 (2%) were blind. There were 132 (1.9%) participants who were unable to report VA; this group included primarily pre-verbal children.

Visual impairment and blindness was significantly more common among individuals over 70 years old (p<0.001).

Ametropias were diagnosed in 5,406 (78.6%) of the individuals who were evaluated by the mobile eye health unit; 660 (9.6%) were diagnosed with cataract and 483 (7%) with pterygium; 247 (3.6%) of the participants had a fully normal exam. Corrective lenses were prescribed for 4,101 (60.9%) of the participants, while 718 (10%) did not require a change in lens prescription; 1908 individuals (28.4%) required only counseling at the time of the visit.

**Figure 2:**
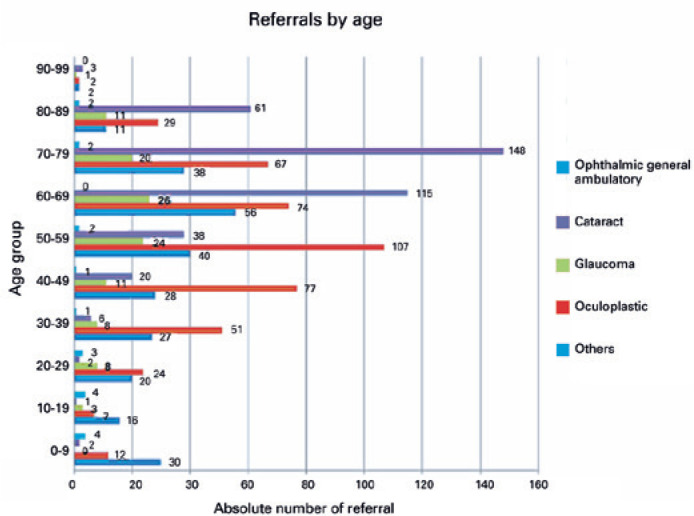
Distribution of referrals to a tertiary hospital for ophthalmic assistance after eye examination in a mobile eye health unit stratified by age

**Figure 3:**
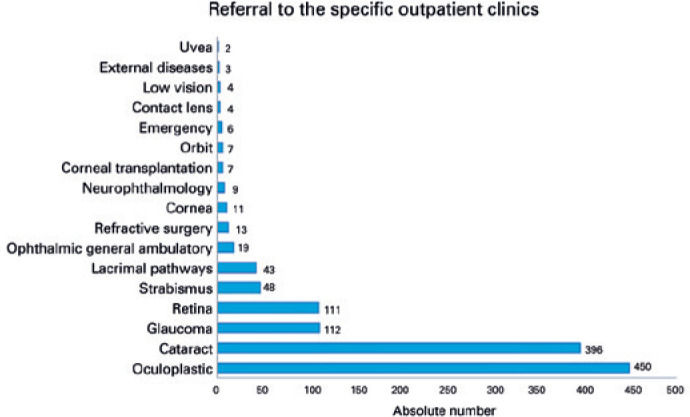
Distribution of ophthalmic subspecialty referrals to a tertiary hospital after eye examination in a mobile eye health unit.

A full 81.7% (5,619 patients) were treated successfully in the mobile eye health unit. Successful treatment was significantly higher among females (83.5%) than among males (78.8%; p=0.03); 1,245 of the participants (18.1%) required referral to a tertiary eye center (Figure 2). Likewise, participants older than 60 years of age were more likely to require referral (p<0.001). Most of referrals were for oculoplastics/external eye or cataract surgery (Figure 3).

Logistic regression analysis revealed that defects in VA correlated with gender (females were more likely to have visual impairment), age (more visual impairment was detected among the elderly), presence of comorbidities (visual impairment was more likely among patients with more comorbidities) and locality of residence (visual impairment was more likely among those living far from specialized centers; Table 3).

**Table 3:** Multiple logistic regression according to gender, age, presence of comorbidity, and residence of each of the participants

Variable	Coefficient (error)	P-value	OR	IC (95%)
Sex	-0.3600 (0.0717)	<0.001	0.697	(0.606;0.803)
Age (years)	0.0490 (0.0022)	<0.001	1.050	(1.046;1.055)
Comorbidities	0.0953 (0.0481)	0.047	1.100	(1.001;1.209)
Residence locality	0.0258 (0.0081)	0.002	1.026	(1.010;1.043)

## DISCUSSION

In the present study, the majority of participants were female. This observation concurs with previous studies from Brazil^(19,20)^. However, this outcome may be different in countries with socio-cultural and economic limitations that reduce women’s access to health care^(4,21)^.

There was no age restriction for participation in this study. As such, we were able to identify characteristics of individuals who required ophthalmic care in the general population. Similar to other surveys, the most common ocular complaints were those related to refractive errors^(19,22,23)^. Other complaints such as headache, pain, hyperemia, tearing and burning sensations can be manifestations of asthenopia; if included as such, this would further increase the complaints related to refractive errors.

Hypertension and diabetes mellitus were the most common comorbidities in this population. However, it is critical to note that these conditions were self-reported condition. The majority of the participants had never presented with eye problems but for those with a previous ocular history, refractive error predominated.

The current study was conducted in the state of São Paulo, which is economically the most developed region in Brazil and has the highest concentration of ophthalmologists^(9)^. Surprisingly, the burden of untreated visual impairment was quite high and similar to that reported in regions with little to no access to health care^(4,6)^. This outcome may relate to the fact that we enrolled individuals who were seeking eye care from the mobile unit; this may have resulted in an overestimation of visual impairment and blindness compared to those members of the community who have routine access to eye care. Hence, our findings may not accurately represent the absolute prevalence of visual impairment and blindness in São Paulo as a whole.

There was a significant increase in the number of blind and visually impaired among the elderly, a finding that confirms those from previous reports^(2-4)^ and clearly reflects the ophthalmic problems related to aging^(24,25)^. These observations indicate the necessity of providing additional assistance to the elderly who require monitoring and ophthalmic care.

Refractive error was the most common condition and was diagnosed in 78.6% of the participants. This outcome also confirms those in previous reports^(10,19,20)^. In the current study, 60.9% of the participants needed a prescription for corrective lenses; these results suggest that these regions require more dispensaries that prepare and fit eyeglasses ^(23)^. Eyeglasses are more common in subjects older than 50 years primarily due to presbyopia^(13)^.

The mobile eye health unit is an efficient method for providing eye care; we found that 81.7% of the participants had their issues resolved in a single visit. In a similar study^(20)^ the resolution rate was 91.1% and the main reason for referral was surgery, similar to the results obtained here. Another, more qualitative study reported a resolution rate of 85.9% as part of a secondary referral service^(26)^, although Covolo et al. reported resolution of only 44.8% of cases in a single visit^(19)^. The differences may relate to different needs that are unique to a specific region and/or issues secondary to the health care infrastructure.

The 18.1% of participants who required referrals were primarily those who needed surgery or additional examination with specialized equipment. The evaluation in the mobile eye health unit in these cases could be considered an important screening service which provided critical referrals to specific outpatient clinics.

The need for referrals to the tertiary service varied between genders. Although the majority of participants were female, the need for referral was statistically higher for males. This finding may indicate males seeking ophthalmic care tend to be those who have more serious pathology; it is certainly possible cultural influences are such that men seek for medical attention only when the pathology is more severe.

The logistic regression analysis revealed that an increased risk of visual impairment was directly correlated with aging, the presence of more comorbidities and distance resided from a tertiary care hospital; any efforts made toward preventive measures should take these factors into consideration. Mobile eye health units working in cooperation with the local health services can be an even more effective screening tool and referral framework if they can focus on overcome these specific barriers^(27)^.

The vast majority of patients presenting to a mobile eye health unit required a prescription for corrective lenses. Mobile eye clinics are highly efficient for managing eye problems; they can prescribe corrective lenses for under-diagnosed refractive errors and refer patients to specialized ophthalmic services as needed. Taken together, our results indicate that mobile eye health units can be used as an effective, alternative method to deliver eye care as they can improve access, provide public health education and ultimately reduce visual impairment and prevent blindness in largely under-served populations.
